# Antimicrobial Activity of Truncated and Polyvalent Peptides Derived from the FKCRRWQWRMKKGLA Sequence against *Escherichia coli* ATCC 25922 and *Staphylococcus aureus* ATCC 25923

**DOI:** 10.3390/molecules22060987

**Published:** 2017-06-14

**Authors:** Nataly de Jesús Huertas, Zuly Jenny Rivera Monroy, Ricardo Fierro Medina, Javier Eduardo García Castañeda

**Affiliations:** 1 Chemistry Department, Universidad Nacional de Colombia, Bogotá Carrera 45 No. 26-85, Building 451, Office 409, Laboratory 334, Bogotá 11321, Colombia; njhuertasm@unal.edu.co (N.d.J.H.); zjriveram@unal.edu.co (Z.J.R.M.); rfierrom@unal.edu.co (R.F.M.); 2 Pharmacy Department, Universidad Nacional de Colombia, Bogotá Carrera 45 No. 26-85, Building 450, Office 203, Bogotá 11321, Colombia

**Keywords:** bovine lactoferricin, antibacterial activity, synthetic peptides, branched peptides, *E. coli*, *S. aureus*

## Abstract

Peptides derived from LfcinB were designed and synthesized, and their antibacterial activity was tested against *Escherichia coli* ATCC 25922 and *Staphylococcus aureus* ATCC 25923. Specifically, a peptide library was constructed by systemically removing the flanking residues (N or C-terminal) of Lfcin 17–31 (^17^FKCRRWQWRMKKLGA^31^), maintaining in all peptides the ^20^RRWQWR^25^ sequence that corresponds to the minimal antimicrobial motif. For this research, also included were (i) a peptide containing an Ala instead of Cys ([Ala^19^]-LfcinB 17–31) and (ii) polyvalent peptides containing the RRWQWR sequence and a non-natural amino acid (aminocaproic acid). We established that the lineal peptides LfcinB 17–25 and LfcinB 17–26 exhibited the greatest activity against *E. coli* ATCC 25922 and *S. aureus* ATCC 25923, respectively. On the other hand, polyvalent peptides, a dimer and a tetramer, exhibited the greatest antibacterial activity, indicating that multiple copies of the sequence increase the activity. Our results suggest that the dimeric and tetrameric sequence forms potentiate the antibacterial activity of lineal sequences that have exhibited moderate antibacterial activity.

## 1. Introduction

Inappropriate use of antibiotics has allowed the emergence of pathogens that cause infections that cannot be resolved using commercially available antibiotics, limiting the possibilities for treatment and cure. The World Health Organization (WHO) has stated that some strains of *E. coli*, *Klebsiella pneumoniae*, *Staphylococcus aureus*, *Streptococcus pneumoniae, Salmonella, Shigella* spp., and *Neisseria gonorrhoeae* have exhibited resistance to conventional treatments [1]. Antimicrobial peptides (AMPs) are considered to be a valuable source for the design and production of new antimicrobial drugs [2,3]. They are part of the innate immune response, have low antigenicity, and exhibit antimicrobial activity against Gram-positive and Gram-negative bacteria, fungi, viruses, and parasites. Furthermore, AMPs are molecules with multiple action mechanisms, a broad activity spectrum, and a low potential for inducing resistance [4,5,6,7,8,9,10,11]. These characteristics imbue them with great potential as therapeutic agents for the treatment of bacterial infections.

Bovine Lactoferricin (LfcinB: ^17^FKCRRWQWRMKKLGAPSITCVRRAF^41^) is a 25-amino acid AMP obtained from bovine lactoferrin protein (BLF) as a hydrolysis product through gastric pepsin action [12,13]. LfcinB exhibits amphipathic α-helix conformation between residues 17–29, shown by circular dichroism in 2,2,2-Trifluoroethanol (TFE). However, it has been established via NMR that LFcinB and LfcinB (17–30) do not adopt the conformation of α-helix, favoring the formation of β-sheets [14,15]. LfcinB has exhibited antiviral, antiparasitic, antifungal, anticancer, and antibacterial activity against Gram-positive and Gram-negative bacteria [16,17,18,19,20,21,22,23,24,25]. In similar way to others AMPs, the suggested action mechanism for LfcinB consists of an initial interaction of an electrostatic nature between the positively-charged amino acid side chains (Arg) and the negative charges of bacterial wall surface molecules (Lipopolysaccharides, teichoic acid) [9,10,21,22,23,24,25,26,27,28,29], and then a second interaction between the side chains of hydrophobic residues (Trp) and the lipid bilayer, leading to membrane destabilization and subsequent cell lysis [18,24,30,31]. However, it has been possible to identify LfcinB, LfcinB 17–31, and D-LfcinB 17–31 in the cytoplasm of *E. coli* ATCC 25922 and *S. aureus* ATCC 25923, suggesting that LficnB can act through other mechanisms [10,27,32,33]. Short synthetic peptides derived from LfcinB have been reported that have displayed antibacterial activity against Gram-positive and Gram-negative bacteria [19,33,34,35,36,37]. The peptide LfcinB 17–31 (^17^FKCRRWQWRMKKLGA^31^) has presented antibacterial activity similar to or higher than LfcinB. This 15-residue peptide is of great interest, since it has exhibited antibacterial activity against *E. coli* (ATCC 25922; K88, L361, O9, ML35), *Salmonella choleraesuis* (CMCC50020), *Salmonella typhimurium* (CMCC50013), *Pseudomonas aeruginosa* (CMCC27853), *S. aureus* (ATCC 25923, 42D), and *Bacillus subtilis* (ATCC 6633) [32,33,35,38,39,40,41]. The sequence RRWQWR has been reported to be the minimal motif showing antibacterial activity [42,43], and this sequence has also exhibited anticancer activity [44]. Previously, it has been reported that synthetic peptides containing the RRWQWR sequence exhibited activity similar to or greater than LfcinB [32,34,36,37,40,45,46,47,48]. It has also been reported that the inclusion of non-natural amino acids at specific sites of the sequence and/or presentation of the sequences in cyclic, dimeric, and tetrameric form increases antibacterial activity [19,36,37,46,49,50,51,52]. In the present article, we report the design and synthesis of lineal, dimeric, and tetrameric peptides derived from LfcinB (17–31) and their antibacterial activity against *E. coli* ATCC 25922 and *S. aureus* ATCC 25923.

## 2. Results and Discussion

We report the design, synthesis and antibacterial activity of 13 peptides derived from LfcinB 17–31. All the peptides contain the minimal motif reported to exhibit antibacterial activity, (RRWQWR) [42,43,53] (Table 1).

Please note that short peptides were designed by sequentially removing N-terminal residues (peptides LfcinB 18–31 to LfcinB 20–31), or C-terminal residues (LfcinB 17–30 to LfcinB 17–25). In addition, also included in this research were (i) a peptide with Ala residue instead of Cys at the nineteenth position ([Ala^19^]-LfcinB 17–31), (ii) polyvalent peptides, specifically a tetramer, LfcinB (20–25)_4_, that was obtained by oxidation of the dimeric peptide (RRWQWR)_2_K-Ahx-C and a dimer of [Ala^19^]-LfcinB 17–31 sequence, and (iii) a palindromic sequence, RWQWRWQWR. As controls, the peptides LfcinB 17–31, LfcinB 20–25, and BLF protein were also included.

**Figure 1:**
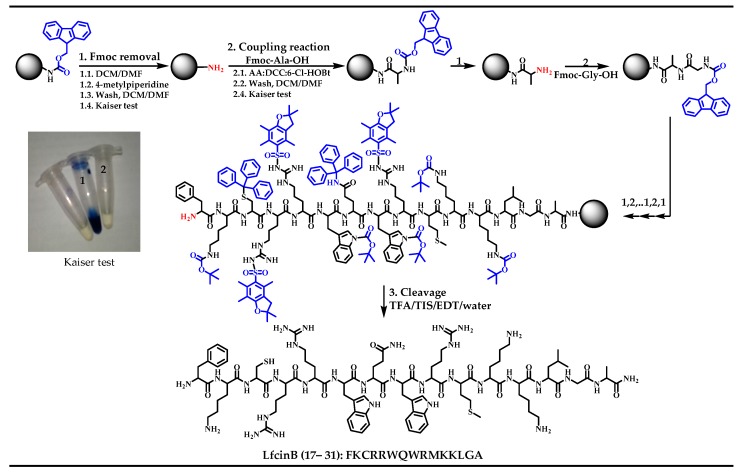
Scheme of the SPPS-Fmoc/tBu. The α-amino and side chains protecting groups are in blue. In the Kaiser Test, (1) amino free detection, after Fmoc removal reaction; and (2) non-amino group detection, after coupling reaction.

The designed peptides were synthesized manually via solid phase peptide synthesis (SPPS) using the Fmoc/tBu strategy (Figure 1). The sequences mainly required only a single coupling reaction for each amino acid residue, indicating that the coupling strategy using *N*,*N*-dicyclohexylcarbodimide (DCC) and 1-hydroxy-6-chlorobenzotriazole (6-Cl-HOBt) was adequate. Our results indicate that these sequences can be obtained via Solid Phase Peptide Synthesis (SPPS) quickly and with good yields (Table 1). However, for all peptides a difficult coupling process in the incorporation of both ^20^Arg and ^21^Arg residues was observed, requiring more time and double the quantity of reagents. This agrees with previous reports, which have shown that the incorporation of amino acids such as cysteine, methionine, arginine, and tryptophan, or sequences having repeating amino acids in several zones, may present difficulties in the synthesis and production of undesired species and require expensive purification processes [54].

**Figure 2:**
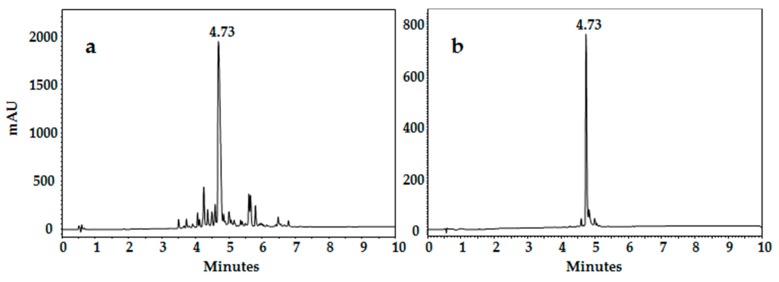
RP-HPLC analysis of Peptide LfcinB 17–25. Chromatographic profile of (**a**) crude and (**b**) purified product.

The crude products were analyzed by RP-HPLC, and in all cases the chromatographic profiles showed a main species (Figure 2a) with a relative area percentage between 44% and 72% (210 nm detection). Yields of the crude product ranged from 60% to 95%, the lowest yielding peptide being LfcinB (17–29). These results are congruent with those obtained for other similar sequences derived from LfcinB [36,55]. It should be noted that all the sequences contain Arg residues, whose protective side group (Pbf) required long removal reaction times (up to 12 h), and it was necessary to use scavengers at high concentrations. In addition, the sequences contain tryptophan residues, whose side chain is highly sensitive to alkylation reactions and, in cleavages with prolonged durations, can form an adduct with the ethanedithiol (EDT) and Trifluoroacetic acid (TFA) [56].

The peptides were purified by means of solid-phase extraction using a C18 cartridge Reversed Phase-Solid Phase Extraction (RP-SPE), and from this process molecules were obtained with purities (determined by RP-HPLC) between 83% and 97%. The overall yield of the pure product ranged from 5% to 37% (Table 1). As an example, Figure 2 shows the chromatographic profile of the peptide LfcinB 17–25 before and after the purification process. Pure peptides were analyzed via MALDI-TOF mass spectrometry, and in all cases a major signal corresponding to the *m*/*z* ratio of the [M + H]^+^ ion was observed, where M is the monoisotopic mass of the desired species (Table 1). Our results indicate that SPPS is versatile, allowing the production of linear, dimeric, or tetrameric peptides, as well as the incorporation of non-natural amino acids.

The development of antibacterial therapeutic agents based on peptides derived from LfcinB is of great interest, since LfcinB and short synthetic peptides have exhibited antibacterial activity against Gram-positive and Gram-negative bacteria, and have also exhibited synergism with antibiotics and antifungal agents, which provides an alternative for the treatment of resistant strains [24]. In the present paper, we evaluated the antibacterial activity of peptides derived from the sequence LfcinB 17–31 (Table 2), which corresponds to a fragment of LfcinB that has been reported to exhibit greater antimicrobial activity than that obtained for LfcinB (25 residues) itself.

It was found that the peptide LfcinB 17–31 and the short peptides LfcinB 17–28, LfcinB 17–29, LfcinB 17–30, and LfcinB 20–31 have an MIC of 200 μM against *E. coli* ATCC 25922, while the peptides LfcinB 19–31, LfcinB 17–25, LfcinB 17–26, and LfcinB 17–27 exhibited higher antibacterial activity (100 μM) than LfcinB 17–31, suggesting that specific changes in the sequence may influence the antibacterial activity against this strain. It can also be deduced that the N-terminal (FKC) and C-terminal (KLGA) amino acids flanking the RRWQWR motif are not critical for antibacterial activity. On the other hand, Peptide LfcinB 17–26 was the only lineal peptide that exhibited antibacterial activity against *S. aureus* ATCC 25923 (MIC and MBC of 200 μM), whereas, the peptide LfcinB 17–31 and its short lineal analogues exhibited no activity at the tested concentrations.

Studies of the antibacterial activity with analogues (scan Ala) of LfcinB peptide 17–31 show that the change of the underlying amino acid, ^17^FKCRRWQWRMKKLGA^31^, causes the loss of the antibacterial activity against *S. aureus* ATCC 25923 compared to the activity of LfcinB 17–31. Additionally, the ^22^Trp and ^24^Trp positions were found to be essential for the antibacterial activity of the peptide LficnB 17–31 against *E. coli* ATCC 25922 [47]. Therefore, it has been suggested that the antibacterial activity of the peptide LfcinB 17–31 against *E. coli* and *S. aureus* proceeds by different mechanisms. This is in agreement with a study showing that LfcinB, L-LfcinB (17–31), and D-LfcinB (17–31) peptides had no post-antibiotic effect against *S. aureus* 25923 and two clinical isolates of *S. aureus*. The authors suggest that this behavior is possibly due to the concentrations used [32]. It was also shown that D-LfcinB had a higher post-antibiotic effect than the L-amino acid containing analogues, suggesting that this effect is due to D-amino acids conferring resistance to the sequence against proteolytic degradation caused by pathogen proteases [32]. The peptide LfcinB 17–25 exhibited higher activity against *E. coli* than the LfcinB peptide 17–31, and the peptide subfragment 1 (FKCRRWQWR-Homoserine lactone) exhibited antibacterial activity against *E. coli* L361 (10 μM), *E. coli* O9 (15 μM), and *S. aureus* (>50 μM) [40], indicating that LfcinB 17–25 is a sequence that exhibits antibacterial activity against different strains of *E. coli*. The antibacterial activity determined for LfcinB 19–31 agrees with that reported previously for this sequence against *E. coli* (120 μM) and *S. aureus* (>150 μM).

The LfcinB 20-31 peptide did not exhibit antibacterial activity against any of the strains tested, however its analogue LfcinB 20–30 has shown antibacterial activity against *E. coli* ML35 (LD99.9; 15 μM) and *S. aureus* 42D (LD99.9; 7.5 μM) [35]. Our results also agree with the reported antibacterial activity (*E coli*, MIC: 252 μM, *S. aureus* MIC: >500 μM) for the sequence SKCYQWQRRMRKLGA. Please note that in this sequence some C-terminal positions have been changed compared to LfcinB 17–31 [38,48]. The peptide LfcinB 17–31 has been studied for its relative structure and antibacterial activity [41]. It has been suggested that ^22^Trp and ^24^Trp residues are important for the antibacterial activity of the peptide, and studies with analogue peptides show that the antibacterial activity of this sequence depends on the size, shape, and aromatic character of the side chains. Specific changes in the residues of tryptophan and cysteine by amino acids containing bulky aromatic groups in the side chain affect antibacterial activity against *E. coli* ATCC 25922 and *S. aureus* 25923 [41].

**Figure 3:**
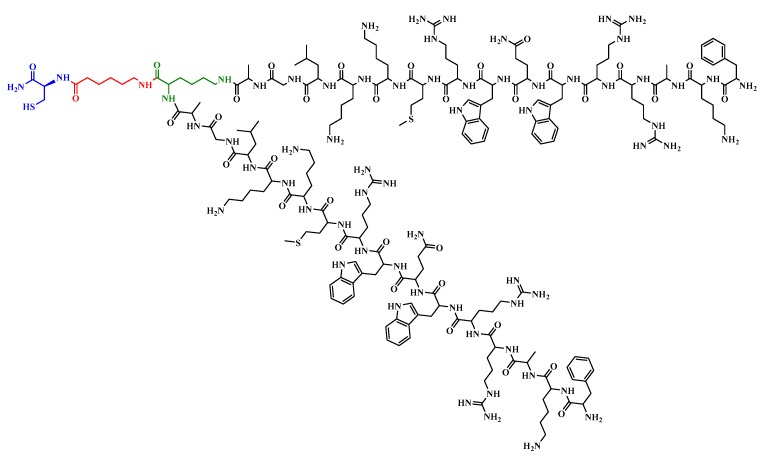
Structure of Polyvalent peptide ([^19^Ala]-LfcinB 17–31)_2_. The structure contains lysine (in green), aminocaproic acid (in red) and cysteine (in blue) residues.

The antibacterial activity against *S. aureus* of both the LfcinB 17–31 and its analogue containing an Ala residue instead of Cys residue, at position 19 ([^19^Ala]-LfcinB 17–31), was >200 μM (Table 2). However, when this sequence was constructed as a dimer ([^19^Ala]-LfcinB (17–31)_2_, Figure 3), it exhibited greater antibacterial activity against this strain (MIC and MBC of 12.5 μM). Table 2 show the MBC result of both [^19^Ala]-LfcinB 17–31 and its dimer, which exhibit higher antibacterial activity than those reported for LficnB 17–31 against *S. aureus* by other authors (MIC; 48 μM, 32 μM, 40 μM, [39,41,57]).

The antibacterial activity of the peptide LfcinB (20–25) was also determined against the two strains, and the MIC and MBC values were found to be greater than 200 μM. This peptide corresponds to the sequence reported by other authors as the minimal motif of activity (RRWQWR) [53]. The antibacterial activity of this sequence was significantly increased (MBC and MIC ≤ 25 μM) when the tetrameric form, (LfcinB 20–25)_4_, was used. Our results agree with the results reported by [58], which showed that the multiple antigen peptides derived from LfcinH sequences exhibited higher antibacterial activity than their linear counterparts. Previous results show that dimeric and tetrameric structures derived from LfcinB containing the RRWQWR motif exhibited greater antibacterial and anti-narcotic activity than linear sequences. The above results indicate that the dimeric and tetrameric forms of LfcinB-derived sequences can enhance the antibacterial activity of promising lineal sequences.

Furthermore, the palindromic peptide LfcinB (21–25)_Pal_ exhibited increased activity against *E. coli* (MIC 6.25 μM and MBC 12.5 μM). This is in accordance with previous results showing that this peptide exhibits antibacterial activity against Gram-positive and Gram-negative bacteria [36,37]. The shape of a palindromic sequence allows the Arg and Trp residues to alternate, forming an amphipathic structure, and this possibly favors the interaction of the peptide with the bacterial membrane [27,38]. Our results are consistent with the fact that some antimicrobial peptides exist as dimers when bound to vesicles (magainin 2 and MS 1-78), which has led to the generation of dimeric peptides covalently bound by disulfide bonds [29]. This strategy has been used to generate parallel and antiparallel dimers of magainin 2 and dimeric forms of AMPs derived from lentiviruses. In both cases, dimerization increased the antibacterial activity [14,49,50,51,52]. Previous studies show that palindromic structures containing Trp and Arg residues have exhibited antibacterial activity against *E. coli*. [19].

The tested peptides listed in Table 2 do not exhibit hemolytic activity at the concentrations that exhibited antibacterial activity against the evaluated strains.

## 3. Materials and Methods

### 3.1. LfcinB-Derived Peptide Synthesis

The designed peptides (Table 1) were synthesized using manual solid-phase peptide synthesis (SPPS-Fmoc/tBu), as is shown in Figure 2 [36,37,55]. Briefly, Rink amide resin (150 mg, 0.46 meq/g) was used as a solid support (Figure 2, Step 1). The resin was treated twice with *N*,*N*-Dimethylformamide (DMF) and Methylene Chloride (DCM), and in order to release the amine group from the resin, it was treated twice for 10 min with the Fmoc removal reagent, 20% 4-methylpiperidine in DMF. Then, the resin was washed with DMF (5×), and DCM (5×) (Figure 2, Step 2). For the coupling reaction, Fmoc-amino acids (0.21 mmol) were pre-activated with DCC/6-Cl-HOBt (0.20/0.21 mmol) in DMF at Room Temperature (RT). The pre-activation mixture was continuously stirred at room temperature (RT) for 15 min. After that, the activated Fmoc-amino acid was added to a reactor containing the deprotected resin and the coupling reaction was stirred for 2 h at RT. Then, the resin was washed with DMF (3×) and DCM (2×). Fmoc group elimination and the incorporation of each amino acid was confirmed by the Kaiser (ninhydrin) test [59] (Figure 2, Step 3). Side chain deprotection reactions and peptide separation from the solid support were carried out with a “cleavage” cocktail containing TFA/water/ Triisopropyl silane (TIS)/EDT (93/2/2.5/2.5% *v*/*v*). The reaction was stirred for 6 h (for some sequences up to 12 h) at RT, and then the mixture was filtered and the solution was collected. Crude peptides were precipitated by treatment of the filtered solution with cold ethyl ether, and finally they were washed with ether five times. The crude products were analyzed by means of RP-HPLC, as will be described in the analytical methods. The tetrameric peptides were obtained by the oxidation of dimeric peptides (RRWQWR)_2_-K-Ahx-C, in accordance with Reference [36]. Briefly, the purified dimeric peptide was treated with DMSO 10% in a buffer of pH 7.5. The oxidation reaction was monitored by RP-HPLC, and it was stopped when the dimer signal disappeared. Then tetramer molecule was desalted using an RP-SPE cartridge.

### 3.2. Analytical Methods

#### 3.2.1. Reverse Phase HPLC

RP-HPLC analysis was performed on a Merck Chromolith^®^ C18 (50 × 4.6 mm) column using an Agilent 1200 liquid chromatograph (Omaha, Nebraska, USA) with UV-Vis detector (210 nm). For the analysis of crude peptides (10 μL, 1 mg/mL), a linear gradient was applied from 5% to 70% Solvent B (0.05% TFA in AcN) in Solvent A (0.05% TFA in water) for 11.5 min at a flow rate of 2.0 mL/min at room temperature.

#### 3.2.2. Peptide Purification

Molecules were purified using solid-phase extraction columns (SUPELCO LC-18 with 2.0 g resin). SPE columns were activated prior to use with 30 mL acetonitrile (containing 0.1% TFA) and equilibrated with 30 mL water (containing 0.1%TFA). Crude peptides were passed through the column, and a gradient was used for their elution. Collected fractions were analyzed using RP-HPLC (as describe above). Fractions that contained pure products were lyophilized.

#### 3.2.3. MALDI-TOF MS

This analysis was performed on an Ultraflex III TOF-TOF mass spectrometer (Bruker Daltonics, Bremen, Germany) in reflectron mode, using an MTP384 polished steel target (BrukerDaltonics), 2,5-dihydroxybenzoic acid, or sinapinic acid (1 mg/mL) as a matrix; Laser: 500 shots and 25–30% power.

### 3.3. LfcinB-Derived Peptide Antibacterial Activity

#### 3.3.1. Antibacterial Activity

The MIC was determined using a microdilution assay [30]. In brief, bacterial strains were incubated for 18 to 24 h at 37 °C in an Müller-Hinton (MH) broth until an optical density of 0.15 to 0.30 (620 nm) was obtained. Next, 90 μL of Mueller-Hinton broth (MHB) was mixed with 90 μL of peptide, and serial dilutions (200, 100, 50, 25, 12.5 and 6.2) were prepared in 96-well microplates (final volume 100 μL/well). Then, 10 μL of inoculum (2 × 10^6^ CFU/mL) was added to each well. The final volume in each well was 100 μL. Then they were incubated for 24 h at 37 °C; the absorbance at 620 nm was measured using an Asys Expert Plus ELISA reader. For determining the MBC, a small sample was taken from each well using an inoculation loop, and then was spread on MH agar plates and incubated overnight at 37 °C. MBC was considered on the plate which presented no bacterial growth. Each of these tests was performed twice (*n* = 2).

#### 3.3.2. Hemolytic Activity Assay

Hemolytic activity was determined in accordance with the methodology described in Reference [44]. Red blood cells (RBCs) from a healthy volunteer (O+), were collected in tubes containing heparin and centrifuged (375 g/15 min). The supernatant was discarded, and the cells were washed several times with 0.9% saline solution. Then the cells were re-suspended in Phosphate buffered saline (PBS) (pH 7.4). An aliquot of 100 μL of RBCs (2% hematocrit) was mixed with 100 μL of the peptide stock solution (concentrations from 50 to 200 μg/mL) and then was incubated for 2 h at 37 °C. The solutions were centrifuged (1150 g/5 min), and the absorbance of each supernatant was measured at 620 nm. This procedure was carried out three times [37,44].

## Figures and Tables

**Table 1:** Designed peptides derived from LfcinB 17–31.

	Code	Sequence	Purified Product		
			Yield (%)	Characterization	
				RP-HPLC	MALDI-TOF
				t_R_ (min)	*(m*/*z)* [M + H]^+^
**Lineal peptides**	LfcinB 17–31	FKC**RRWQWR**MKKLGA	16	4.98	1994.71
	LfcinB 18–31	KC**RRWQWR**MKKLGA	10	4.71	1849.12
	LfcinB 19–31	C**RRWQWR**MKKLGA	25	4.99	1718.00
	LfcinB 20–31	**RRWQWR**MKKLGA	9	4.95	1617.38
	LfcinB 17–30	FKC**RRWQWR**MKKLG	12	4.96	1922.48
	LfcinB 17–29	FKC**RRWQWR**MKKL	5	5.06	1865.73
	LfcinB 17–28	FKC**RRWQWR**MKK	5	4.68	1752.55
	LfcinB 17–27	FKC**RRWQWR**MK	13	4.87	1625.18
	LfcinB 17–26	FKC**RRWQWR**M	7	5.17	1497.06
	LfcinB 17–25	FKC**RRWQWR**	11	4.73	1365.82
	LfcinB 20–25	**RRWQWR**	37	4.19	986.66
	[Ala^19^]-LfcinB 17–31	FKA**RRWQWR**MKKLGA	20	4.94	1961.99
**Polyvalent peptides**	([Ala^19^]-LfcinB 17–31)_2_	(FKA**RRWQWR**MKKLGA)_2_K-Ahx-C	18	5.52	4255.51
	(LfcinB 21–25)_Pal_	**RWQWR**WQWR	30	5.95	1488.58

**Table 2:** Antibacterial activity of designed peptides derived from LfcinB 17–31.

Code		*E. coli*		*S. aureus*	
		ATCC 25922		ATCC 25923	
		MIC	MBC	MIC	MBC
**Lineal peptides**		**200**	**>200**	**>200**	**>200**
		>200	>200	>200	>200
		**100**	**100**	>200	>200
		200	>200	>200	>200
		200	200	>200	>200
		200	>200	-	-
		200	200	>200	>200
		**100**	200	>200	>200
		**100**	200	**200**	**200**
		**100**	**100**	>200	>200
		>200	>200	>200	>200
		-	-	>200	>200
**Polyvalent peptides**		-	-	**12.5**	**12.5**
	(LfcinB 21–25)_Pal_	**12.5**	**25**	-	-
	(LfcinB 20–25)_4_	**6.25**	**12.5**	**25**	**25**
**Protein**	BLF	>50	>50	>50	>50

*n* = 2; Minimum Inhibitory Concentration (MIC) and Minimum Bactericidal Concentration (MBC) in μM.
